# A Case of Primary Hepatic Mucosa-Associated Lymphoid Tissue Lymphoma in a Patient With Primary Biliary Cholangitis and Hashimoto's Thyroiditis

**DOI:** 10.7759/cureus.65472

**Published:** 2024-07-26

**Authors:** Yunlong Li, Liangrui Zhou, Ning Zhang, Yuchen Wei, Chen Yang, Xiaohong Liu, Lin Kang

**Affiliations:** 1 Department of Internal Medicine, Peking Union Medical College Hospital, Peking Union Medical College, Chinese Academy of Medical Sciences, Beijing, CHN; 2 Department of Pathology, Peking Union Medical College Hospital, Peking Union Medical College, Chinese Academy of Medical Sciences, Beijing, CHN; 3 Department of Geriatrics, Peking Union Medical College Hospital, Peking Union Medical College, Chinese Academy of Medical Sciences, Beijing, CHN; 4 Department of Hematology, Peking Union Medical College Hospital, Peking Union Medical College, Chinese Academy of Medical Sciences, Beijing, CHN

**Keywords:** hypothyroidism, management, hashimoto's thyroiditis, primary biliary cholangitis, mucosa-associated lymphoid tissue (malt) lymphoma

## Abstract

Mucosa-associated lymphoid tissue (MALT) lymphoma is a low-grade malignant lymphoproliferative disease, representing a low percentage of newly diagnosed lymphoma cases. Although its exact cause is still unclear, it is commonly associated with infections or autoimmune diseases. The stomach is the most frequent site for MALT lymphoma, with primary hepatic MALT lymphoma being exceptionally rare. Cases of primary hepatic MALT lymphoma often coincide with viral hepatitis. In this report, we present a case of primary hepatic MALT lymphoma in a patient with no history of hepatitis but complicated by primary biliary cholangitis (PBC) and Hashimoto's thyroiditis.

## Introduction

Mucosa-associated lymphoid tissue (MALT) lymphoma, classified as a low-grade, indolent non-Hodgkin lymphoma (NHL), is the most prevalent type of marginal zone lymphoma (MZL) [1]. Common sites affected by MALT lymphoma include the stomach, intestines, lungs, thyroid, and salivary glands. The development of MALT lymphoma may be associated with infections or autoimmune conditions. *Helicobacter pylori* infection is recognized as a major contributor to primary gastric MALT lymphoma, while chronic inflammation from conditions such as Sjögren's syndrome and Hashimoto's thyroiditis can elevate the risk of salivary gland and thyroid MALT lymphoma [1-3]. Although primary hepatic MALT lymphoma is exceedingly rare, with fewer than 130 reported cases, some patients with this condition have a history of long-term hepatitis B virus (HBV) or hepatitis C virus (HCV) infections, highlighting the potential role of infections in MALT lymphoma pathogenesis [4,5]. The influence of autoimmune diseases in this context remains uncertain. While only a small number of reported cases of primary hepatic MALT lymphoma are associated with autoimmune diseases such as primary biliary cholangitis (PBC) and autoimmune hepatitis (AIH), this article presents a case of primary hepatic MALT lymphoma in a patient without hepatitis B or hepatitis C virus infection but with PBC and Hashimoto's thyroiditis. The aim is to enhance clinicians' understanding of this rare disease.

## Case presentation

A 60-year-old female presented at the hospital in January 2024 with intermittent periumbilical swelling and pain. The abdominal pain was not associated with meals but subsided after passing gas or having a bowel movement. She denied experiencing fever, diarrhea, nausea, vomiting, blood in the stool, melena, weight loss, or weight gain. The patient had a history of invasive ductal carcinoma of the right breast (T2N1M0, stage IIb) diagnosed in March 2011. She underwent a right radical mastectomy with axillary lymph node dissection, followed by six cycles of postoperative epirubicin+paclitaxel chemotherapy and local radiotherapy. In 2018, a liver cyst was identified but left untreated, with regular follow-ups. Hashimoto's thyroiditis was diagnosed in July 2023, for which the patient alternately took levothyroxine 50/37.5 μg every day (qd), along with alpha-calciferol 0.25 μg qd and vitamin D 5000 U qd for postmenopausal osteoporosis. The patient, retired for five years, had no history of smoking or drinking. Apart from the patient's father who had cardiac cancer, there was no family history of cancer or hereditary diseases. At diagnosis, the patient had a BMI of 22.6 kg/m^2^. A palpable 1 cm lymph node was noted on the right side of the neck, with no other peripheral lymph nodes palpable. Heart and lung auscultations were normal, and the abdomen was soft with non-palpable liver and spleen.

**Figure 1:**
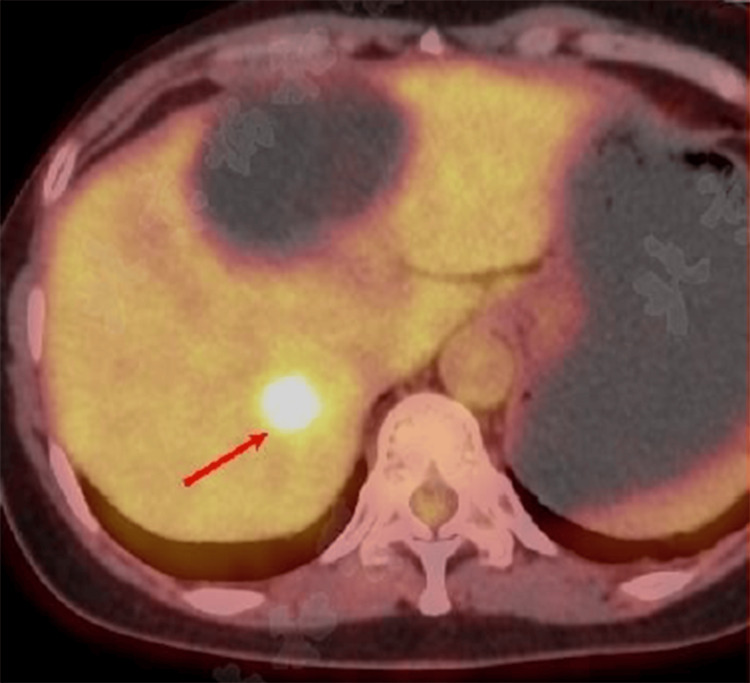
18F-FDG PET/CT imaging identified a 2.5×2.3 cm hyperplastic focus in the right lobe with a SUVmax of 9.3 (arrow) 18F-FDG, 18F-fluorodeoxyglucose; SUVmax, maximum standardized uptake value

**Figure 2:**
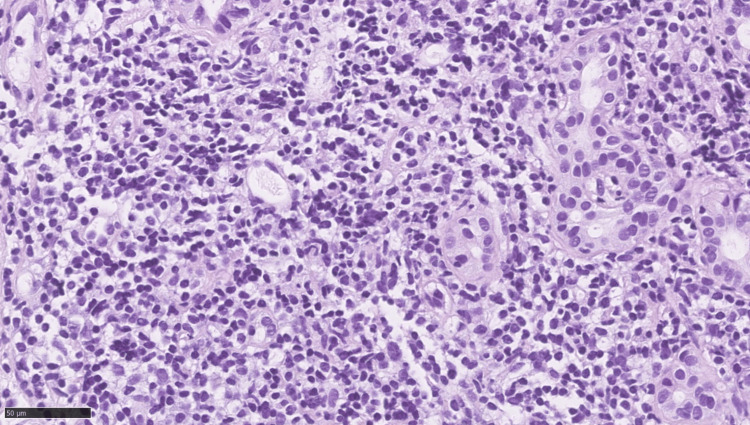
Biopsies confirmed the absence of cancer cells in the left lobe cyst but detected lymphocytic infiltration in the right lobe tumor

In February 2024, the patient underwent an enhanced abdominal CT scan, which showed a 2.5 cm low-density shadow in the right lobe of the liver and a 7.3 cm cystic low-density shadow in the left lobe. Subsequent enhanced MRI scans revealed abnormal signals in both lobes, suggesting potential malignancy in the right lobe and a possible cystadenoma in the left. PET/CT imaging identified a 2.5×2.3 cm hyperplastic focus in the right lobe with a maximum standardized uptake value (SUVmax) of 9.3 (Figure 1). The patient tested negative for hepatitis B surface antigen (HBsAg) and anti-HCV antibodies, ruling out past viral hepatitis. Autoantibody screening revealed positive antinuclear antibodies (reference negative) and antimitochondrial antibodies (AMA)(+) of 1:320 (reference: <1:80) and AMA-M2(+) of >400 U/mL (reference: <20 U/mL), indicating primary biliary cholangitis, which led to the initiation of ursodeoxycholic acid treatment. An extremely high level of anti-thyroglobulin (anti-Tg) antibodies (305.3 IU/mL; reference: <4.5 IU/mL) and antithyroid peroxidase (anti-TPO) antibodies (>1300 IU/mL; reference: <60 IU/mL) corresponded with Hashimoto's thyroiditis. The mass in the right lobe was diagnosed as primary liver cancer, leading to hepatic arterial chemoembolization with 10 mL of lipiodol and 30 mg of epirubicin hydrochloride and subsequent microwave ablation. Biopsies confirmed the absence of cancer cells in the left lobe cyst but detected lymphocytic infiltration in the right lobe tumor (Figure 2). Immunohistochemistry showed B-cell lymphoma antigen (Bcl) 2(+), Bcl-6(+), cluster of differentiation (CD) 3(+), CD5(+), CD10(+), CD20(+), CD21 (follicular dendritic cells {FDC}+), CD23(+), cyclin D1(-), Ki-67 (index 3%), SRY-box transcription factor 11 (SOX11)(-), CD19(+), and lymphoid enhancer-binding factor 1 (LEF-1)(-), which supported a diagnosis of MALT lymphoma. PET/CT findings ruled out lesions elsewhere, confirming primary liver lymphoma. The patient underwent bone marrow puncture and digestive endoscopy simultaneously, with no lymphoma involvement detected in the bone marrow smear and biopsy. Additionally, no abnormalities were observed in the stomach and colon. Both the 13C breath test and gastric *Helicobacter pylori* rapid urease test (HP-RUT) yielded negative results. Following the interventional treatment, a liver MRI re-examination in May 2024 showed no new lesions, prompting the patient to opt for regular follow-up observations.

## Discussion

MALT lymphoma is a low-grade, indolent non-Hodgkin lymphoma [1]. According to the fifth edition of the WHO Classification of Hematolymphoid Tumors (WHO-HAEM5) [6], MALT lymphoma falls under the category of MZL and is the most common pathological type within this group. The gastrointestinal tract, particularly the stomach, is the primary site affected by MALT lymphoma, accounting for about 50% of cases [1]. Apart from the gastrointestinal tract, MALT lymphoma can also involve organs such as the parotid gland, thyroid, lungs, eyes, and skin. Primary liver MALT lymphoma is exceptionally rare, with only 123 reported cases to date [7-17], including 118 cases in English literature and five cases in Chinese literature (as shown in Table 1) since Isaacson et al. first described it in 1995 [18].

**Table 1:** Clinical characteristics of primary hepatic MALT lymphoma (including this case) The numbers in brackets meant the range of matched characters NA, not available; PBC, primary biliary cholangitis; AIH, autoimmune hepatitis; MALT, mucosa-associated lymphoid tissue

	Cases (N=123)
Median age (year)	62 (30-89）
Sex	
Male	57
Female	65
NA	1
Liver disease	
Hepatitis B	40
Hepatitis C	13
PBC	7
AIH	2
Treatment	
Surgery	33
Radiotherapy	2
Chemotherapy	23
Radiofrequency ablation	3
Surgery+chemotherapy	22
Observation	8
NA	32
Median follow-up time (months)	23 (1-111）
Outcome	
Disease-free survival	80
Relapse	10
Death	6
NA	27

The pathogenesis of MALT lymphoma remains unclear, with a general belief that the disease may be linked to chronic inflammation or infection at the primary site [2,3]. For instance, gastric MALT lymphoma could be associated with chronic gastritis following *Helicobacter pylori* infection, while intestinal MALT lymphoma might be related to *Campylobacter jejuni* infection. MALT lymphomas originating in the parotid gland and thyroid gland are respectively linked to chronic inflammation caused by autoimmune diseases such as Sjögren's syndrome and Hashimoto's thyroiditis. Some studies have shown that certain patients with hepatic MALT lymphoma also have complications such as viral hepatitis B or C, PBC, and AIH. The case we presented did not involve HBV or HCV infection but included PBC and Hashimoto's thyroiditis. This represents the first reported case of primary hepatic MALT lymphoma combined with two autoimmune diseases. However, it is important to note that a significant number of patients with primary liver MALT lymphoma do not have infections or chronic inflammation, suggesting the involvement of other mechanisms in the disease's development. Early cases dating back to 1997 highlighted IgH rearrangement and t(3:14)(q27:q32) chromosome translocation in the patients with primary liver MALT lymphoma [19], indicating a potential role of genetic factors. Subsequent studies revealed t(14:18)(q32:q21) chromosome translocation and the trisomy of chromosomes 3 and 8, although their specific molecular mechanisms remain undisclosed [4,20].

Xu et al. found that more than 50% of the patients with primary hepatic MALT lymphoma are asymptomatic [5], with normal serum tumor markers. These patients are frequently discovered incidentally during routine physical examinations or while being evaluated for other health issues. Primary hepatic MALT lymphoma often manifests as hypoechoic nodules on ultrasound, with abdominal CT scans revealing a low-density mass in the liver that may show minimal or no enhancement. MRI scans typically exhibit a low T1 signal and a high T2 signal, while 18F-FDG PET/CT scans suggest slightly increased uptake. FAPI PET/CT scans, on the other hand, show more pronounced uptake compared to 18F-fluorodeoxyglucose (18F-FDG) PET/CT scans [9]. The non-specific nature of imaging tests can sometimes lead to the misdiagnosis of primary liver MALT lymphoma as primary liver cancer, especially in patients with underlying chronic liver disease [5]. The patient was initially thought to have primary liver cancer but was later confirmed to have primary liver MALT lymphoma through a needle biopsy during treatment. Pathology is considered the gold standard for diagnosing primary hepatic MALT lymphoma. While fine-needle aspiration biopsy guided by CT or ultrasound is less invasive, it has a low positive rate. Surgical resection, although offering treatment and the confirmation of the diagnosis, carries a relatively high risk. The pathological examination of MALT lymphoma typically shows a dense proliferation of small lymphocytes that can infiltrate mucosal epithelium to form lymphoepithelial lesions [1]. Despite displaying non-specific immunohistochemistry markers such as CD3, CD5, CD10, Bcl-2, CD19, CD20, CD21, and CD23, a definitive diagnosis can only be made after ruling out other small B-cell lymphomas.

Primary hepatic MALT lymphoma is a rare condition, and as a result, there is a lack of standardized treatment approaches or recommended clinical trials. From the literature we examined, as shown in Table 1, it was found that the majority of the patients (N=33) underwent surgical treatment, chemotherapy (N=23), or a combination of both (N=22). However, a small number of patients chose follow-up observation (N=8), local radiotherapy (N=2), or interventional treatment (N=3). Chemotherapy protocols commonly used for follicular lymphoma, such as rituximab-based regimens such as rituximab, cyclophosphamide, doxorubicin hydrochloride, vincristine (Oncovin), and prednisone (R-CHOP), are considered suitable. Despite the chosen treatment modality, the prognosis for primary liver MALT lymphoma is generally positive, with most patients (N=80) not experiencing disease progression during follow-up. In this specific case, the patient initially underwent two interventional treatments and is planned for follow-up observation in the future. Ursodeoxycholic acid will be continued for the treatment of PBC, while chemotherapy is not currently part of the treatment plan.

## Conclusions

Primary hepatic lymphoma is extremely rare and often misdiagnosed as primary liver cancer, particularly in patients with chronic liver disease. Biopsy could help to make a definitive diagnosis for patients with atypical clinical characteristics. Up to now, the optimal treatment for primary hepatic lymphoma is still unknown, and patients should be followed up closely.
